# Enhanced Uptake of Fe_3_O_4_ Nanoparticles by Intestinal Epithelial Cells in a State of Inflammation

**DOI:** 10.3390/molecules22081240

**Published:** 2017-07-27

**Authors:** Gang Zhou, Jin Zhang, Chun Pan, Naicheng Liu, Zhenheng Wang, Junfeng Zhang

**Affiliations:** 1 State Key Laboratory of Pharmaceutical Biotechnology, School of Life Sciences, Nanjing University, No. 163, Xianlin Avenue, Qixia District, Nanjing 210023, China; gzhou@aliyun.com (G.Z.); ZJ940701@126.com (J.Z.); 18352538639@126.com (C.P.); 2 School of Medicine, Nanjing University, No. 22, Hankou Road, Gulou District, Nanjing 210093, China; liunaicheng@suda.edu.cn (N.L.); wangzhenheng1987@163.com (Z.W.)

**Keywords:** inflammation, Caco-2 cell monolayers, nanoparticles, uptake

## Abstract

Fe_3_O_4_ nanoparticles (Fe_3_O_4_ NPs) have been used for medical and drug applications, although the mechanisms of cellular uptake and transport need to be further evaluated under inflammatory conditions. In the present study, we investigated the uptake of Fe_3_O_4_ NPs (20, 50, 100, and 200 nm) by intestinal epithelial cells under inflammatory conditions via the light scattering of flow cytometry and inductively coupled plasma mass spectrometry (ICP-MS) techniques. The results of the correlation analysis indicated that the uptake ratios of Fe_3_O_4_ NPs by intestinal epithelial cells under inflammatory conditions were higher than those under the control conditions. The transportation ratios of NPs by inflammatory Caco-2 cells increased almost 0.8–1.2 fold compared to the control. The internalization of the Fe_3_O_4_ NPs in Caco-2 cells was mediated by clathrin-related routes in both the control and an interleukin-1β (IL-1β)-induced inflammatory condition. The level of mRNA of clathrin expressed in Caco-2 cells that were stimulated by IL-1β was almost three times more than the control. Consistently with the mRNA expression, the level of protein in the clathrin was upregulated. Additionally, it was verified for the first time that the expression of clathrin was upregulated in IL-1β-stimulated Caco-2 cells. Collectively, these results provided a further potential understanding about the mechanism of Fe_3_O_4_ NPs’ uptake by intestinal epithelial cells under inflammatory conditions.

## 1. Introduction

Over the past few years, the nanoparticle-based drug delivery system has been most widely used as a suitable vehicle for overcoming the pharmacokinetic limitations associated with conventional drug formulations [1,2,3,4,5]. To be effective, the nanoparticles (NPs) must transport packages across various biological barriers that limit the migration of foreign particles. The intestinal epithelial barrier makes the delivery of NPs inefficient and difficult [6,7,8]. Therefore, it is necessary to fully understand the mechanism in addition to the efficiency of uptake and transportation of NPs across the intestinal epithelial barrier.

Until now, a number of in vitro studies have investigated the effect of variable components, surface character, or particle size properties of NPs on their cellular uptake and transportation [9,10,11,12]. However, the process of NPs across the intestinal epithelial barrier is quite complicated [13,14,15]. Furthermore, inflammation plays an important role in the dysregulated function of an epithelial barrier. Under inflammatory conditions, there will be changes in the protein complexes consisting of transmembrane proteins that interact extracellularly with adjacent cells and intracellularly with adaptor proteins, which bind to the cytoskeleton [16,17,18]. Few studies have fully reported the related uptake and transport mechanisms of NPs in intestinal epithelial cells under inflammatory conditions. These may be of the greatest importance for the further study of the epithelial barrier’s function as well as for the uptake and transport of NPs in the state of inflammation. Although the culture model of Caco-2 cell monolayers is widely used to assess the uptake and transport mechanisms of NPs across the intestinal epithelial barrier, there are no suitable in vitro models of the intestinal barrier in the state of inflammation. Different combinations of proinflammatory stimuli (lipopolysaccharides, interleukin-1β, and interferon-γ) and intestinal epithelial cell lines (Caco-2, HT-29, and T84) were evaluated by some researchers. Only Caco-2 cells were responsive to stimulation, with interleukin-1β being the strongest stimulator [19,20].

In the present study, we used Caco-2 cell monolayer models stimulated by IL-1β and animal models chemically induced by dextran sulfate sodium salt (DSS) to study the uptake mechanism that is related to the process of crossing intestinal epithelial cells [21,22]. Having utilized the light scattering of flow cytometry [23,24] and ICP-MS techniques, higher uptake ratios of Fe_3_O_4_ NPs by Caco-2 cell monolayers under inflammatory conditions were verified compared to those under control conditions. Internalization of the Fe_3_O_4_ NPs was mediated by clathrin-related routes under both control and inflammatory conditions. For the first time, the upregulated expression of clathrin in IL-1β stimulated Caco-2 cells was verified. In short, this work provides a further potential understanding of the mechanism and efficiency of Fe_3_O_4_ NPs’ uptake by intestinal epithelial cells under inflammatory conditions.

## 2. Results and Discussion

### 2.1. Characterization of NPs

**Figure 1:**
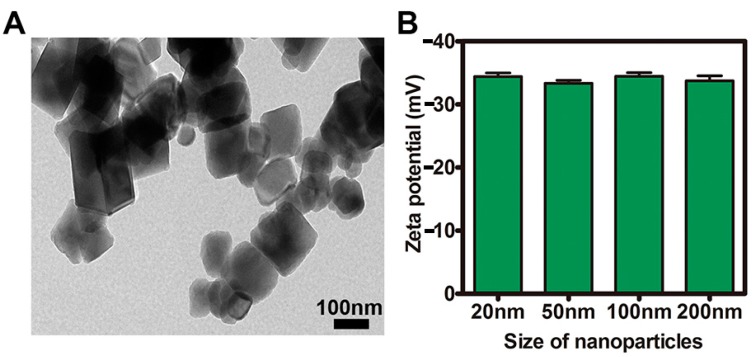
Characterization of nanoparticles (NPs) (**A**) Transmission electron microscopy (TEM) image of Fe_3_O_4_ NPs; (**B**) The surface charge of NPs.

The TEM image and the negative zeta potentials of the NPs are presented in Figure 1A,B. The surface charge zeta potentials of the NPs were determined using the zeta potential analyzer at a concentration of 500 μg/mL in aqueous solution. As shown in Figure 1A, Fe_3_O_4_ NPs had a uniform morphology with some agglomerates. Figure 1B indicates the negative charge of the NPs at physiological pH.

### 2.2. CCK-8 Assays and Measurement of Transepithelial electrical resistance (TEER) and Inflammation

**Figure 2:**
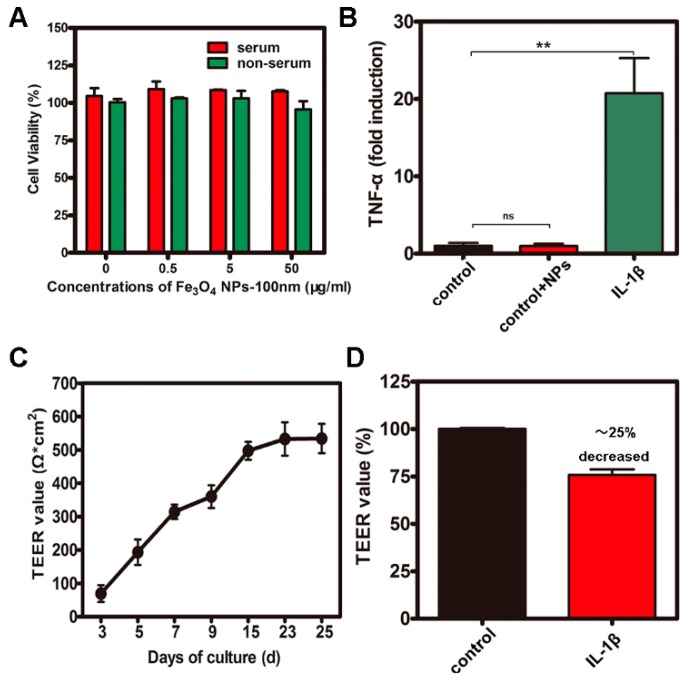
CCK-8 assays as well as the measurements of transepithelial electrical resistance (TEER) and inflammation of Caco-2 cell monolayers. (**A**) Cytotoxicity assay of cells after treatment with Fe_3_O_4_ NPs; (**B**) The level of mRNA of TNF-α expression with IL-1β stimulation in Caco-2 cells; (**C**) The monolayer with a TEER value higher than 550 Ω·cm^2^ after 25 days in culture; (**D**) The decrease of TEER value of Caco-2 cell monolayers with IL-1β stimulation. *ns =* not-significant, ** *p* <0.01.

The results (Figure 2A) collectively approved the high biocompatibility of Fe_3_O_4_ NPs. The threshold concentration (50 μg/mL) was used in all subsequent experiments. Transepithelial electrical resistance (TEER) is a technique to measure the integrity of tight junction dynamics in cell culture models of epithelial monolayers. The Caco-2 monolayer generates a TEER of 150–400 Ω·cm^2^, which limits the diffusion of substances across the barrier. Figure 2C shows that the monolayer (cultured for 25 days) had a TEER value that was higher than 550 Ω·cm^2^. After inducing inflammation, the IL-1β stimulation in the Caco-2 cells increased the mRNA of TNF-α expression up to more than 20-fold compared to the control (Figure 2B). Figure 2D shows that the structural change in the tight junctions results in a modest decrease in TEER of about 25%.

### 2.3. Tissue Distribution and Cellular Localization of Fe_3_O_4_ NPs

**Figure 3:**
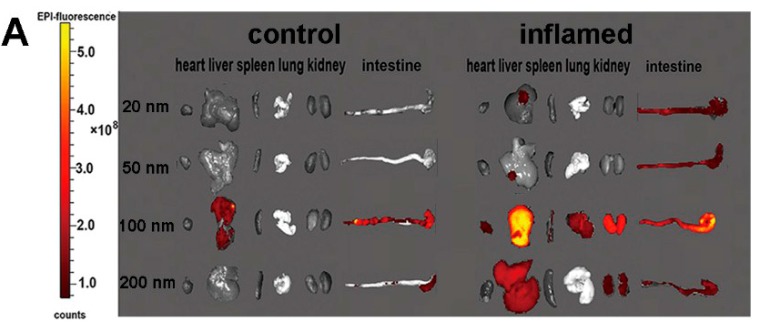

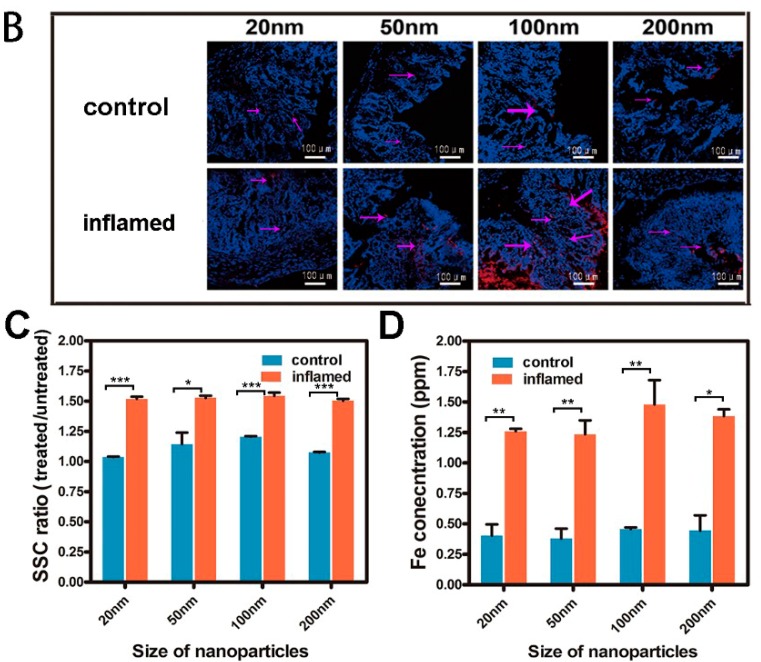
Tissue distribution of Fe_3_O_4_ NPs and the uptake of the Fe_3_O_4_ NPs in intestinal epithelial cells. (**A**) Fluorescence imaging of the different organs of mice; (**B**) The localized of Fe_3_O_4_ NPs in intestinal epithelial cells. (Red color and purple arrow, Fe_3_O_4_ NPs; blue, DAPI nuclear staining); (**C**) The side scatter (SSC) ratio reflecting the NPs in the cells was investigated by light scattering with flow cytometry; (**D**) The amount of Fe_3_O_4_ NPs in the cells was investigated by ICP-MS. * *p* <0.05, ** *p* <0.01, and *** *p* < 0.001.

Figure 3 shows that the concentration of Fe_3_O_4_ NPs in different organs from mice at 3 h, after Fe_3_O_4_ NPs were slowly administered into the lumen of the colon. All of the results from the fluorescence images indicated that inflammation enhanced the accumulation of Fe_3_O_4_ NPs in relevant organs. Meanwhile, the results showed that Fe_3_O_4_ NPs with a size of 100 nm accumulated in relevant organs more than those NPs with other sizes. The results in Figure 3B indicate that the amount of Fe_3_O_4_ NPs localized in inflammatory intestinal epithelial cells is more than that localized in controlling intestinal epithelial cells. Consistently with the Figure 3A images, 100 nm Fe_3_O_4_ NPs were significantly increased in an inflamed colon. The uptake of the Fe_3_O_4_ NPs in intestinal epithelial cells was quantitatively investigated by light scattering with flow cytometry and ICP-MS. Figure 3C,D displays the amount of Fe_3_O_4_ NPs in mouse intestinal epithelial cells. The uptake of the Fe_3_O_4_ NPs increased significantly in mouse intestinal epithelial cells under inflammatory conditions (3% DSS-induced) compared with control.

### 2.4. Investigation of the Uptake of Fe_3_O_4_ NPs in Caco-2 Cells

**Figure 4:**
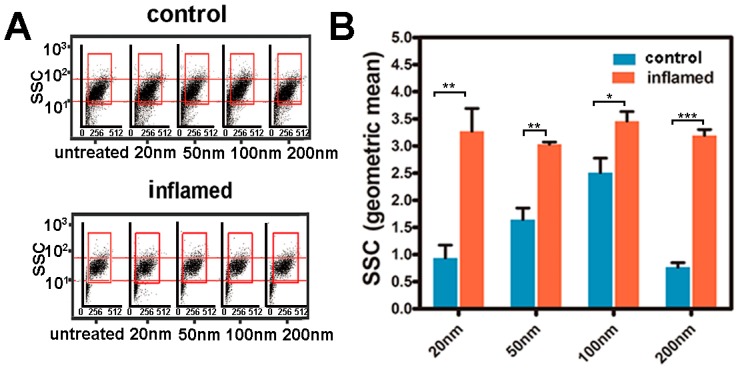

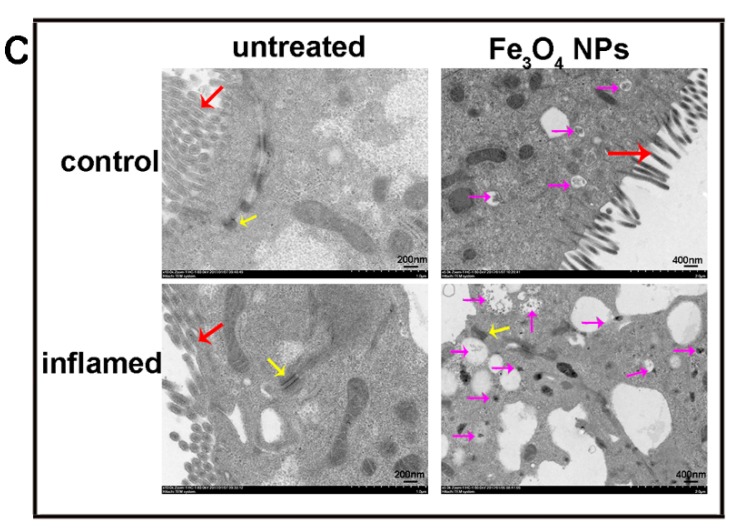
The uptake of Fe_3_O_4_ NPs in Caco-2 cells. (**A**) Flow cytometry light scattering plots of Caco-2 cells treated with Fe_3_O_4_ NPs; (**B**) Amount of Fe_3_O_4_ NPs uptake by Caco-2 cells (The SSC reflecting the NPs in cells); (**C**) TEM images for the Fe_3_O_4_ NPs in Caco-2 cells. (Red arrow = microvillus; yellow arrow = tight junctions; purple arrow = Fe_3_O_4_ NPs). * *p* <0.05, ** *p* <0.01, and *** *p* <0.001.

The results in Figure 4A,B that were measured by light scattering with flow cytometry illustrate the uptake of the Fe_3_O_4_ NPs in Caco-2 cells under inflammatory conditions compared with control. According to Figure 4A,B, there was a significant increase in the side scatter (SSC) of Caco-2 cells under inflammatory conditions. In addition, Figure 4B indicates that the maximum Fe_3_O_4_ NPs uptake by Caco-2 cells occurs at 100 nm with sizes from 20 to 200 nm. For further study of the uptake of the Fe_3_O_4_ NPs in Caco-2 cell monolayers, the ultrastructure of the cells and NPs in Caco-2 cell monolayers was observed by TEM. Figure 4C shows the integrality of Caco-2 cell monolayers and the decrease in microvillus under inflammatory conditions. The decrease of microvillus may provide nanoparticles with more opportunities for contact with the cell membrane. The integral structure between cells was consistent with the TEER value. These results indicate that nanoparticles found it difficult to cross the Caco-2 cell monolayers by the paracellular pathway. Obviously, the amount of Fe_3_O_4_ NPs in Caco-2 cells under inflammatory conditions was greater compared with other control groups (Figure 4C).

### 2.5. Investigation of the Uptake Features of Fe_3_O_4_ NPs in Caco-2 Cells

**Figure 5:**
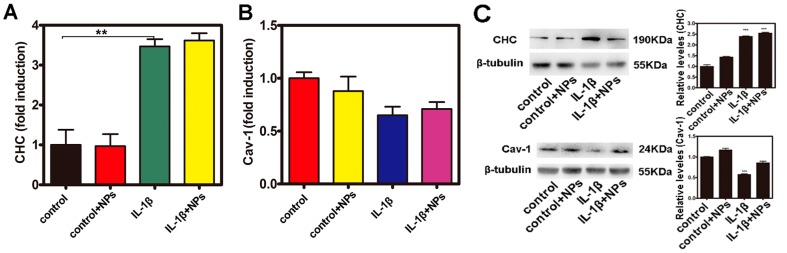

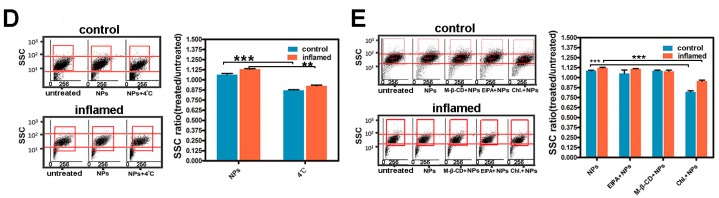
Investigation of the uptake features of Fe_3_O_4_ NPs in Caco-2 cells. (**A**,**B**) The level of mRNA expression of clathrin heavy chain (CHC) and Cav-1; (**C**) The level of protein expression of CHC and Cav-1 detected by Western blotting (WB); (**D**,**E**) Impacts of various uptake inhibitors on the uptake of Fe_3_O_4_ NPs, quantitative amounts of remaining NPs in the cells. ** *p* <0.01, and *** *p* <0.001.

Cellular endocytosis can be divided into clathrin-mediated endocytosis, caveolae-mediated endocytosis, macropinocytosis, and phagocytosis [25]. Owing to the excellent clathrin- and caveolae-mediated uptake ability of NPs by cells [26,27], in this study, the levels of mRNA as well as the protein levels of clathrin and caveolae were assayed in Caco-2 cells. As shown in Figure 5A, the level of mRNA of clathrin expressed in Caco-2 cells that were stimulated by IL-1β was almost three times more than the control. Consistently with mRNA expression, the protein level of clathrin was upregulated when measured by Western blotting (WB) (Figure 5C). However, as shown in Figure 5B,C, the level of mRNA and protein of caveolae was not upregulated in Caco-2 cells with IL-1β stimulation. In addition, Caco-2 cells were maintained at 4 °C to study whether cellular uptake of the Fe_3_O_4_ NPs was an energy-dependent process. Furthermore, several pathways have been reported for the endocytosis of nanoparticles into cells, including the macropinocytosis pathway and clathrin- and caveolae (or lipid raft)-related routes. M-β-CD, which can bind cholesterol, exerts an effective inhibition of the caveolae (or lipid raft)-mediated endocytosis pathway. Chloropromazine, which blocks the assembling of clathrin at the cell membrane, is often used to inhibit the clathrin-mediated route. EIPA has been reported to effectually inhibit the macropinocytosis-mediated route. Figure 5D,E shows that the uptake of the Fe_3_O_4_ NPs in Caco-2 cells was significantly inhibited at 4 °C or with chloropromazine treatment both under control and inflammatory conditions.

### 2.6. Transport of the Fe_3_O_4_ NPs in Caco-2 Cell Monolayers

**Figure 6:**
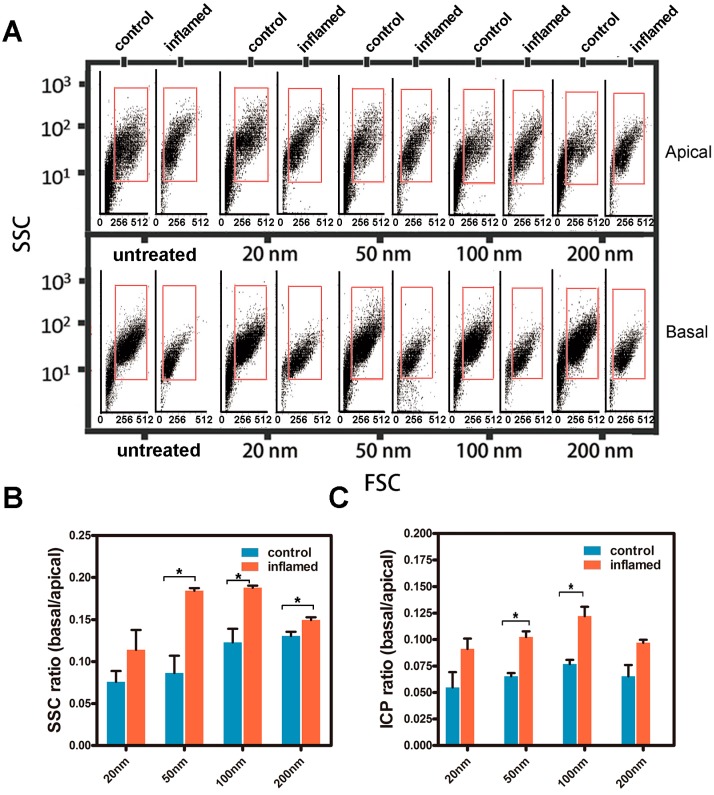
Transport of the Fe_3_O_4_ NPs in a Caco-2 cell monolayer. (**A**) Flow cytometry light scattering plots of the forward scatter (FSC) and the SSC of Caco-2 cells treated with Fe_3_O_4_ NPs in a trans-well system; (**B**,**C**) The transport of the Fe_3_O_4_ NPs in a Caco-2 cell monolayer was quantitatively investigated using the light scattering with flow cytometry and ICP-MS methods, respectively. * *p* < 0.05.

To further study the uptake and transport of the Fe_3_O_4_ NPs in Caco-2 cell monolayers, the specimens on the apical or basal side after 12 h of transportation were analyzed. Light scattering plots from flow cytometry of the forward scatter (FSC) and side scatter (SSC) of Caco-2 cells cultured in a trans-well system after the transportation of NPs are shown in Figure 6A. According to Figure 6B,C, there was an increase in transportation under inflammatory conditions compared to the control. Figure 6B shows that the transportation ratio of inflammatory cells increased about 0.8–1.2 times compared to the controls. Figure 6C also shows that the transportation ratio (9.1–12.2%) under inflammatory conditions was higher than the transportation ratio (5.5–7.7%) in the controls.

In the present study, we focused on the uptake of Fe_3_O_4_ NPs by intestinal epithelial cells under inflammatory conditions. The intestinal tract is the primary physiological barrier for the oral absorption of multiple substances [28,29]. Lehr et al. [30,31] reported that in the inflamed colon, an increased adherence of particles was observed in the thicker mucus layer. A size dependency of the deposition was found, with an increased number of attached particles to the colon found compared with the control group. The interaction between NPs and cells has gained great attention from scientists in the field of medicine, biology, and material science. Nevertheless, until now, a comprehensive understanding of the entire uptake and transport process as well as the mechanism of NPs across the intestinal tract has been lacking, especially under inflammatory conditions. In addition, NPs may be taken up in cells via different pathways, which may be specific or non-specific and energy-dependent or energy-independent [7,32]. As a result, the uptake of NPs in cells including epithelial cells may be complicated and diverse, resulting in many mysteries still needing to be unraveled. Some in vitro studies have investigated the effect of size or surface properties of NPs on their cellular uptake or transport [33,34]. It has been shown that clathrin-mediated endocytosis was an uptake process, which occurs through the formation of clathrin-coated vesicles that form from the plasma membrane [26]. Clathrin-mediated endocytosis was involved primarily in the internalization of receptors [31,35,36]. However, under inflammatory conditions, there are changes in protein complexes consisting of transmembrane proteins that interact in the extracellular environment with adjacent cells and in the intracellular environment with adaptor proteins that link to the cytoskeleton.

In our study, we used light scattering with flow cytometry and ICP-MS to illustrate the uptake of Fe_3_O_4_ NPs in intestinal epithelial cells in vivo and Caco-2 cells in vitro. Figure 3 showed that the uptake of the Fe_3_O_4_ NPs increased significantly in mouse intestinal epithelial cells under inflammatory conditions compared with the controls. Figure 4B showed that there was a significant increase in SSC of Caco-2 cells under inflammatory conditions. Figure 4C indicated that the amount of Fe_3_O_4_ NPs in Caco-2 cells under inflammatory conditions was greater compared with the controls. Figure 4B indicated that the maximum amount was 100 nm of Fe_3_O_4_ NPs by Caco-2 cellular uptake both under control and inflammatory conditions. The results were mainly consistent with the results from Chan and co-workers [30,37]. Figure 6B showed that the transportation ratio of NPs by inflammatory cells increased 0.8–1.2 fold compared with the control. Figure 6C also showed that the transportation ratio of NPs (9.1–12.2%) under inflammatory condition was higher than the control transportation ratio (5.5–7.7%). In further studies, the levels of mRNA as well as the protein levels of clathrin and caveolae in Caco-2 cells were assayed. The results in Figure 5 showed that the level of mRNA of clathrin in Caco-2 cells that were stimulated by IL-1β was almost 3-fold greater than the controls. Consistently with the mRNA levels, the protein level of clathrin was also upregulated when measured by WB detection.

## 3. Materials and Methods

### 3.1. Materials

Fe_3_O_4_ NPs, 5-(*N*-ethyl-*N*-isopropyl) amiloride (EIPA), methyl-beta-cyclodextrin (M-β-CD), chlorpromazine hydrochloride (Chl.), dextran sulfate sodium salt (DSS), IL-1β, and rhodamine B (RB) were purchased from Sigma-Aldrich (St. Louis, MO, USA). The cell counting kit-8 (CCK-8) was purchased from Dojindo Laboratories (Tokyo, Japan). Caco-2 cell was purchased from the Institute of Biochemistry and Cell Biology (Shanghai, China). Dulbecco’s modified Eagle’s medium (DMEM) and fetal bovine serum (FBS) were obtained from Gibco (Grand Island, NY, USA). The four- to six-week old C57/B6 mice were purchased from the experimental animal center of Nanjing University (Nanjing, China). All other chemicals were analytical grade reagents and were used as received without further purification.

### 3.2. Characterization of NPs

Fe_3_O_4_ NPs of various sizes (20 nm, 50 nm, 100 nm, and 200 nm) were obtained from Sigma-Aldrich (St. Louis, MO, USA). RB-Fe_3_O_4_ NPs were synthesized according to a published method. The amide RB, carbodiimide (EDC), and N-hydroxysuccinimide (NHS) were added to Fe_3_O_4_-PAA solution and stirred for 45 min. The precipitate was separated from the solution with a 0.6 T magnetic field, and washed with water until the supernatant became a colorless solution. After this, the RB-Fe_3_O_4_ NPs were dispersed in phosphate buffer saline (PBS).

To prevent aggregation, the nanoparticles were suspended in filtered deionized water and sonicated. Transmission electron microscope (TEM; Hitachi H-7000FA, Hitachi Ltd., Tokyo, Japan) and zeta potential analyzer (90Plus, Brookhaven Instruments Corp., Holtsville, NY, USA) analyses were used to characterize the size, morphology, and surface charge of the NPs at a concentration of 500 μg/mL in aqueous solution.

### 3.3. Cell Culture

Caco-2 cells were maintained in the DMEM supplemented with 10% FBS and were incubated at 37 °C in a humidified atmosphere of 5% CO_2_ in air in the incubator. To study the uptake process of the NPs in Caco-2 cells, the cells were passaged by a treatment of 0.1% trypsin and 0.02% ethylene diamine tetraacetic acid (EDTA) in PBS, then the cells were seeded in a 12-well sterile plate at a concentration of 10^5^ cells/mL. All of the cells used in this study were between passages 40 and 60. For studying the transport procedure of NPs in the Caco-2 cell monolayers, the cells were seeded on a polycarbonate insert membrane of a trans-well device (Costar Transwell, Millipore Corp., Bedford, MA, USA). A total of 0.5 mL of DMEM containing 2 × 10^5^ cells was seeded in the upper compartment of each well of the trans-well device, while 1.5 mL of cell-free or cell-containing DMEM was poured into the lower compartment. During the long-term culture of 25 days, the mediums in both the upper and basilar compartments were changed every other day, and the transepithelial electrical resistance (TEER) was measured at the same time by epithelial volt-Ω m (Millipore Company, Billerica, MA, USA) to monitor the integrity of the cell monolayers. A total of 5 ng/mL IL-1β was added to the Caco-2 cell monolayers in order to induce the inflammation, which was conducted 3 h before the nanoparticles were added.

Colitis was induced by the addition of DSS to drinking water. The mice were randomly divided into groups (five mice in one group). This study was carried out in strict accordance with the recommendations in the guide for the care and use of laboratory animals of the National Institutes of Health. The protocol was approved by the committee on the ethics of animal experiments of Nanjing University (2016NZGKJ-093). All efforts were made to minimize the suffering in the mice. Mice were treated with dextran sulfate sodium salt solution (DSS-3%) (3%, *w*/*v* in drinking water) for 5 days. The untreated control animals received drinking water only. Mouse intestinal epithelial cells were isolated from the mice according to conventional protocols. The intestine was removed from the mesenteric fat tissue and the colon was opened longitudinally and cut into pieces. The colon was then cut into small fragments and washed with hanks’ balanced salt solution (HBSS) repeatedly until the HBSS remained clear. After incubation with EDTA and dithiothreitol (DTT) in HBSS, the colon segments were then incubated for 2–3 h at 37 °C with 25 mL of digestion mixture (75 U/mL Collagenase XI (Sigma, St. Louis, MO, USA), 20 μg/mL dispase (Roche, Basel, Switzerland), and 1% FBS in DMEM. After digestion, centrifuging, and passing through a cell strainer, the suspension of epithelial cells was collected.

### 3.4. Cell Viability Assay

After the applied treatment, cell viability was examined by CCK-8 assays as per the manufacturer’s protocol. The absorbance at wavelengths of 415 nm and 630 nm was recorded. The effect of NPs on cells was expressed as the percentage of cell viability compared with relative proliferation rate (RPR), which was calculated using the following formula: RPR (%) = (A − AN)/AN × 100%, where A represents the absorbance of each different concentration group and AN is the absorbance of negative control group.

### 3.5. Bio-Distribution and Cellular Location of NPs

RB-Fe_3_O_4_ NPs in PBS solutions were slowly administered into the lumen of the colon via a catheter inserted at a point 4 cm into the colon through the anus. The heart, liver, spleen, lung, kidney, and colon were excised from the treated mice after 3 h. These were photographed by an IVIS Lumina XR system (PerkinElmer, Waltham, MA, USA). The frozen colon sections were incubated with nuclei staining 4,6-diamidino-2-phenylindole (DAPI; Sigma, St. Louis, MO, USA). The frozen colon sections images were captured on a Nikon fluorescent microscope equipped with a digital camera (TE2000-U, Nikon, Tokyo, Japan).

### 3.6. Quantitative Analysis of the Internalized NPs in Intestinal Epithelial Cells

NPs (20, 50, 100, 200 nm) in PBS solutions were slowly administered into the lumen of the colon via a catheter (−1.2 mm in diameter) inserted at a point 4 cm into the colon through the anus. The mouse intestinal epithelial cells were collected after 3 h, and analyzed by light scattering with flow cytometry and PerkinElmer SCIEX ELAN 9000 ICP-MS (PerkinElmer SCIEX, Concord, ON, Canada). A BD FACS Calibur^TM^ (BD Biosciences, San Jose, CA, USA) flow cytometer containing a 488-nm laser, forward scatter diode detector, and photomultiplier tube-side scatter detector was used in this study. Generally, the FSC indicates the overall size of cells, while the SSC provides information about internal structures and organelles. The cytometer was set to measure SSC logarithmically and FSC linearly. The Caco-2 cells were incubated with defined NPs suspensions for 12 h. A total of 500 μL of cell solution (10,000 cells) was used for the light scattering with flow cytometry analysis. In a separate assay, an ICP-MS-based quantification of nanoparticles uptake into cells was used. The cells (counted about 100,000 cells) were acid digested, and after removing any undigested substrate the supernatant was diluted and the metal content was analyzed by ICP-MS.

The endocytosis pathway of NPs was investigated by the addition of specific pharmacological inhibitors for specific cellular uptake pathways as described in Table 1. Prior to the addition of NPs, Caco-2 cells that were grown on the plate were pre-incubated with inhibitors for 1 h at 37 °C. During the nanoparticles’ incubation, the concentration of inhibitors was maintained at a constant value. After the co-incubation of nanoparticles and inhibitors, cellular uptake was aborted by cold PBS. Using rinse and fixation, the cells were examined by light scattering with flow cytometry.

### 3.7. Quantitative Analysis of the Fe_3_O_4_ NPs Transport across Caco-2 Cell Monolayers

The Caco-2 cell monolayers cultured in the trans-well devices were incubated with 50 μg/mL of Fe_3_O_4_ NPs for 12 h. The transport medium in both compartments and Caco-2 cells were then collected and analyzed by ICP-MS. In a separate experiment, the Caco-2 cells in both compartments were then collected and analyzed by light scattering with flow cytometry.

### 3.8. Western Blotting

The cells were lysed in a RIPA lysis buffer (Beyotime, Nanjing, China). Thirty micrograms of each protein were separated by 8% or 10% sodium dodecyl sulphate-polyacrylamide gel electrophoresis (SDS-PAGE), and then transferred to a polyvinylidene fluoride membrane (Pall Co., East Hills, NY, USA). Western blotting was performed using these primary antibodies: Clathrin heavy chain (CHC, Cell Signaling Technology, Beverly, MA, USA, #2410), (dilution ratio: 1:1000) or caveolin 1 (CAV-1, Cell Signaling Technology, Beverly, MA, USA, #3238), (dilution ratio: 1:1000). We incubated the membrane and the primary antibody in 10 mL of primary antibody dilution buffer overnight at 4 °C. Following this, we washed this solution three times for 5 min each with 15 mL of tris-buffered saline and tween 20 (TBST). After this, we probed with a specific primary antibody and incubated with a horseradish peroxidase (HRP)-conjugated goat anti-rabbit secondary antibody (Jackson ImmunoResearch Laboratories, West Grove, PA, USA, #111-005-003), (dilution ratio: 1:2000, 1 h incubation at room temperature). We washed three times for 5 min each with 15 mL of TBST. The protein bands were detected.

### 3.9. Quantitative Real-Time PCR

The total RNA from the Caco-2 cells were prepared using Trizol reagent (Invitrogen, Carlsbad, CA, USA). One microgram of total RNA was reverse transcribed to cDNA with the first-strand cDNA synthesis kit (Invitrogen, Carlsbad, CA, USA). For mRNA detection, the real-time polymerase chain reaction (PCR) was launched in an ABI 7300 Fast Real-time PCR System (Applied Biosystems, FosterCity, CA, USA) using the SYBR Prime Script RT-PCR Kit (Takara Bio, Shiga, Japan). Cyclicparameters of qPCR: initial denaturation temperature was 95 °C for 5 min followed by 40 cycles of 15 s denaturation at 95 °C temperature, 30 s annealing at 60 °C temperature and 45 s extension at 72 °C temperature. Primers of β-actin, TNF-α, CHC, and CAV1 used were listed as follows (Invitrogen, Carlsbad, CA, USA):

- β-actin sense: 5′-GGTGTGATGGTGGGAATGGG-3′;
- β-actin antisense: 5′-ACGGTTGGCCTTAGGGTTCAG-3′.
- TNF-α sense: 5′-CCCAGGGACCTCTCTCTAATCA-3′;
- TNF-α antisense: 5′-AGCTGCCCCTCAGCTTGAG-3′.
- CHC sense: 5′-TGAGGCGACTGGGCGGAGTT-3′;
- CHC antisense: 5′-CCGGGGACGCAGGAAACTGG-3′.
- CAV-1 sense: 5′-GCCAACTACCAGCGTGAC-3′
- CAV-1 antisense: 5′-ATGCCCGCACCTGAGTAA-3′

### 3.10. Transmission Electron Microscopy

The cells were fixed with 2.5% glutaraldehyde in 0.1 M sodium dihydrogen phosphate. The samples were then fixed in 1% OsO_4_ for 1 h, dehydrated by graded ethanol solutions, and gradually infiltrated with epoxy resin. Ultra-thin sections were obtained and stained with uranyl acetate and lead citrate, before being observed in a TEM.

### 3.11. Statistics

All of the results were expressed as the mean value ± standard deviation from at least three independent measurements. The statistical analysis was performed with the two tailed student’s *t*-test, with the statistical significance assigned at *p* <0.05. The corresponding markers in the figures were defined as * *p* <0.05, ** *p* <0.01, and *** *p* <0.001, respectively. 

## 4. Conclusions

In summary, our studies revealed the uptake of Fe_3_O_4_ NPs by intestinal epithelia cells and Caco-2 cell monolayers under inflammatory conditions. The efficiency of the uptake of Fe_3_O_4_ NPs by Caco-2 cell monolayers under IL-1β stimulated inflammatory conditions was significantly higher than the controls because of the upregulation of clathrin. Furthermore, the clathrin-mediated uptake of NPs may be size-dependent both under control and inflammatory conditions.

## Figures and Tables

**Table 1:** Inhibitors with different functions used in the pathway study and their concentrations.

Inhibitors	Functions	Concentrations
EIPA	Inhibitor of endocytosis pathway through macropinocytosis	20 μM
Methyl-beta-cyclodextrin	Inhibitor of lipid raft/caveolae dependent endocytosis	10 mM
Chloropromazine	Inhibitor of clathrin-related route	30 μM
